# A health partnership to reduce neonatal mortality in four hospitals in Rwanda

**DOI:** 10.1186/s12992-017-0252-6

**Published:** 2017-06-01

**Authors:** Placide Ntigurirwa, Kathy Mellor, Daniel Langer, Mari Evans, Emily Robertson, Lisine Tuyisenge, Alan Groves, Tom Lissauer

**Affiliations:** 1 Muhima Hospital, Kigali, Rwanda; 2 BirthLink, Ickenham, UB10 8HN UK; 3 Epsom & St Helier University Hospitals NHS Trust, Surrey, UK; 4 Great Ormond Street Hospital, London, UK; 5 Royal Devon and Exeter Hospital, Exeter, UK; 6 University Teaching Hospital of Kigali, Kigali, Rwanda; 7 Weill Cornell Medicine, New York, USA; 8 Imperial College Healthcare Trust, London, UK; 9 Centre for International Child Health, Imperial College London, Norfolk Place, London, W2 1PG UK

**Keywords:** Global health partnership, Low-income country, Newborn, Infants, Capacity building, Quality improvement, Nutrition, Continuous positive airways pressure, Medical records, Rwanda

## Abstract

**Background:**

A health partnership to improve hospital based neonatal care in Rwanda to reduce neonatal mortality was requested by the Rwandan Ministry of Health. Although many health system improvements have been made, there is a severe shortage of health professionals with neonatal training.

**Methods:**

Following a needs assessment, a health partnership grant for 2 years was obtained. A team of volunteer neonatologists and paediatricians, neonatal nurses, lactation consultants and technicians with experience in Rwanda or low-income countries was assembled.

A neonatal training program was provided in four hospitals (the 2 University hospitals and 2 district hospitals), which focused on nutrition, provision of basic respiratory support with nasal CPAP (Continuous Positive Airway Pressure), enhanced record keeping, thermoregulation, vital signs monitoring and infection control. To identify if care delivery improved, audits of nutritional support, CPAP use and its complications, and documentation in newly developed neonatal medical records were conducted. Mortality data of neonatal admissions was obtained.

**Results:**

Intensive neonatal training was provided on 27 short-term visits by 10 specialist health professionals. In addition, a paediatric doctor spent 3 months and two spent 6 months each providing training. A total of 472 training days was conducted in the neonatal units.

For nutritional support, significant improvements were demonstrated in reduction in time to initiation of enteral feeds and to achieve full milk feeds, in reduction in maximum postnatal weight loss, but not in days for regaining birth weight. Respiratory support with bubble CPAP was applied to 365 infants in the first 18 months. There were no significant technical problems, but tissue damage, usually transient, to the nose and face was recorded in 13%. New medical records improved documentation by doctors, but nursing staff were reluctant to use them. Mortality for University teaching hospital admissions was reduced from 23.6% in the 18 months before the project to 21.7%. For the two district hospitals, mortality reduced from 10% to 8.1%. A major barrier to training and improved care was low number of nurses working on neonatal units and staff turnover.

**Conclusion:**

This health partnership delivered an intensive program of capacity building by volunteer specialists. Improved care and documentation were demonstrated. CPAP was successfully introduced. Mortality was reduced. This format can be adapted for further training and improvement programs to improve the quality of facility-based care.

## Background

In September 2000, global political leaders adopted the United Nations Millennium Declaration, committing to a series of Millennium Development targets including improving health. Millennium Development Goal 4 (MDG 4) focused on reducing <5 years mortality. In Rwanda, a target was set to reduce child mortality from 152/1000 live births in 1990 to 50/1000 live births by 2015 [1]. By 2010 under 5 years mortality was still 76/1000 live births, with neonatal mortality (< 28 days old) comprising 27/1000 live births, and the reduction in neonatal mortality was slower than that in older children.

From 2010, a partnership in child health was in place in Rwanda to provide ETAT+ (Emergency Triage, Assessment and Treatment plus Admission) courses for the recognition and initial management of sick children (Imperial College London, subsequently Royal College of Paediatrics and Child Health, UK and Rwanda Pediatric Association, Rwandan Ministry of Health and Kenya Paediatric Association health partnerships). This well-established, evidence-based, 5-day course covers the 10 commonest causes of paediatric admissions in East Africa [2]. Although the ETAT+ course has a neonatal component, a more extensive neonatal training program was requested by Dr. Agnes Binagwaho, former Rwandan Minister of Health (personal communication, TL).

In Rwanda, there are just over 400,000 births/year [3]. Over 90% of births occur in health centres or district hospitals. Rwanda has under 0.45 healthcare workers per thousand population [4], far below the minimum level recommended by WHO of 2.3 per 1000. There were only about 26 paediatricians and no neonatologists or specialist neonatal nurses in the country.

The aim of this partnership was to improve neonatal care and reduce neonatal mortality in 4 major neonatal units, including the 2 University hospitals and 2 District hospitals, one of which was the largest maternity unit in the country and the other a medium-sized district hospital. After identifying their major training requirements, we aimed to provide a program of intensive capacity building by visiting volunteer health professionals. In order to determine if this resulted in changes in service delivery, several audits were conducted. Our experience would determine if this partnership model could be adopted for future partnerships.

## Methods

### Needs assessment

A detailed needs assessment of neonatal care in the four hospitals was performed. In addition to a detailed inventory of facilities, equipment, investigations and drugs, the training needs were determined by a team of UK neonatologists and neonatal nurses. This was achieved by visiting the neonatal units, attending medical wards rounds and direct observation of medical and nursing care. The units varied in size. The University hospitals each had 12 incubators, 3 radiant warmers, 9 cots and 5 beds for 24-h Kangaroo Mother Care (KMC). Although relatively well equipped with incubators and radiant warmers, there was a shortage of beds for mothers to stay and conduct KMC. The district hospitals had 3–10 incubators, 2–8 radiant warmers and 15 cots. One hospital had 8 beds for KMC, the other initially had none. Only 3 of the units had a functioning oxygen saturation monitor. Each unit was supervised by a paediatrician, but most medical care in the University hospitals was provided by recently qualified doctors or postgraduate trainees; in the district hospitals by junior doctors also covering other specialties. Nurse staffing was very limited, ranging from 10 to 16 for each unit, with 2 to a maximum of 4 nurses per shift. There was no formal neonatal training programme for either doctors or nurses. The major needs identified were:

i) **Nutrition** – to overcome long delays in the early introduction of breast milk feeds to preterm infants. This was because of physical separation of mother and infant (as the mother was in the maternity unit) and lack of advice and help in initiating expression of breast milk, together with reluctance by staff to begin and advance enteral milk feeds in preterm infants due to concerns that the babies were too small to tolerate feeds and fear of risk of serious gastrointestinal complications (necrotising enterocolitis).
ii) **Provision of basic respiratory support** - no unit was able to provide this for infants with respiratory distress, although this is standard care in high-income countries. Bubble CPAP (Continuous Positive Airway Pressure) was considered to be the most pragmatic way to provide this.
iii) **Enhanced Record Keeping** - hospital medical records were unsuitable for neonatal care and data was often missing or difficult to retrieve. Introduction of focused neonatal-specific records was considered a priority to support structured clinical assessments and to allow monitoring of improvements in care [5].
iv) **Thermoregulation** - to improve temperature regulation of preterm and sick infants with appropriate use of available incubators and increase use of KMC.
v) **Vital Sign Monitoring** – to increase access to monitoring of oxygen saturation and vital signs and improve staff’s appreciation of their interpretation.
vi) **Infection Control** – improve hand hygiene, which was compounded by lack of hand basins with running water and hand gel.

### Health partnership grant

In conjunction with the Rwanda Pediatric Association and Rwandan Ministry of Health, a grant was obtained, funded by the United Kingdom Department for International Development (DfID) and managed by the Tropical Health & Education Trust (THET). The primary aim was reduction in neonatal mortality in the 4 hospitals. The grant was for US$45,000 over 2 years, which clearly constrained the size of the project.

### Newborn care training and service improvement program

Core issues identified in the needs assessment were addressed with a newborn care training program. A team of neonatologists, neonatal nurses, lactation consultants and technicians with experience of teaching and training, and willing to participate in the program on a voluntary basis was assembled. Multiple short visits of intensive training were organized, as well as three long-term placements, one 3-months and two 6-months, by paediatric doctors with neonatal experience. A local lead clinician was appointed at each hospital to lead and co-ordinate the program; in 3 hospitals it was the paediatrician, in one district hospital it was a nurse on the unit.

To enhance nutritional support, training was provided emphasising the importance of supporting mothers to provide early and frequent breast milk expression and not to wait until mothers were well enough after delivery to come to the neonatal unit. Training by lactation consultants and neonatal nurses was mainly conducted directly with staff on the maternity and neonatal units, supplemented by lectures and demonstrations. Posters, mainly diagrammatic, but with any text translated in English, French or Kinyarwanda, for mothers and staff were produced to demonstrate techniques and importance of expressing breast milk for preterm infants. Posters were distributed on maternity and neonatal units. Systems for cleaning and sterilising milk expressing equipment, and safe storage of expressed breast milk were introduced.

Respiratory support was provided by bubble CPAP, using a simple system of nasal CPAP in widespread use in Europe and North America (Fisher Paykel), except that air was provided by a small compressor which was part of the system instead of from a wall supply. The system was driven by air, with additional oxygen provided as required. It included an oxygen analyser to allow the oxygen concentration to be monitored. The circuit generated heated humidified gases that were delivered to the infant by nasal cannulae or face mask. The CPAP pressure was regulated by bubbling the gases through a bottle of water. An oxygen saturation monitor suitable for continuous monitoring in newborn infants was provided with each machine. Instead of each patient receiving a new CPAP circuit, as in high-income countries, circuits were washed and re-used to reduce cost and help overcome supply difficulties. As CPAP was new to all the units, and machine availability limited, guidelines were developed to maximise their potential benefit by targeting preterm infants with difficulty breathing and a birth weight 1.0–2.5 kg, and to encourage rapid weaning when breathing improved.

To overcome the difficulty in using the standard hospital medical records, a new neonatal medical record booklet was designed, in consultation with local health professionals and hospital directors. The record consists of structured collection of initial data on antenatal and perinatal health, clinical assessment and management followed by multiple, specially designed, daily summary pages for medical and nursing records and for recording of vital signs as well as fluid input/output charts. Growth charts are also included. After piloting, booklets for the University hospitals were printed. Development of the notes, staff induction and a 3-week training period with feedback was conducted at the University hospitals.

Temperature regulation was problematic as many different makes of incubator were in use, and their efficacy was variable. Demonstrations were provided to optimise their use, and increased use of KMC encouraged.

Vital signs monitoring was improved by the provision of extra oxygen saturation monitors and guidance on their interpretation.

Infection control was improved by practising hand washing technique.

### Audits and data collection

To determine if the training program resulted in changes in clinical practice, 3 detailed audits were conducted. The nutrition audit was led by visiting long-term paediatric doctors (ER and ME), in conjunction with local doctors and nurses. Data from a random sample of records of babies of birthweight <2.5 kg on 3 of the units before and after training were extracted: time to initiation of enteral feeds, time to full milk feeds, maximum postnatal weight loss (percentage of birthweight) and number of days for birthweight to be regained.

The audit of respiratory support with CPAP was led by a Rwandan paediatrician (PN). CPAP use and complications were recorded using a checklist which staff completed on a daily basis.

The audit of completeness of documentation of medical records was led by the long-term visiting paediatric doctors (DL, ME, ER). Medical records were selected at random before and after introduction of the new version and completeness assessed according to a check-list.

Mortality data of infants admitted to the neonatal units, both before and during the project, is collected by the hospital every month and was checked with entries in the unit admission books.

### Statistical methods

Analysis of data from the nutrition audit was with Graphpad Prism statistical software version 7.0. Two-tailed Mann-Whitney statistical analysis was used to compare before and after training results except for data for the day of life enteral feeds were started as this had a normal distribution and so unpaired two-tailed t- test was used.

### Ethical approval

Ethical approval was not required as audits were conducted for quality assurance and data was anonymised. Mortality data is collected routinely for the Ministry of Health.

## Results

### Training provided

The frequency and duration of training provided over the two years of the project is shown in Table 1. Between February 2012 and January 2014, 27 short-term visits by a team of 10 specialist health professionals from the UK were made, most making multiple visits. They provided a total of 252 training days. The medical technicians assisted in setting up the CPAP machines and with initial training. Visits were mostly short and intensive, lasting 1–2 weeks. This was because of difficulty in staff obtaining leave from their hospitals in the UK, with visits generally taken as annual rather than professional leave. Visits were frequent to reinforce the training. Training was provided to 84 Rwandan health professionals on CPAP. The one paediatric doctor who each spent three months and the two who spent six months in Rwanda provided an additional 220 days of training. A major limitation was the small number of nursing staff available for training, with only 2–4 nurses working each shift. In addition, medical and nursing staff often rotated to other departments and to other hospitals.

**Table 1:** Number of health professionals. visits and training days

Health professionals	No. people	No. visits	Training days provided
Neonatologists	3	6	63
Neonatal nurses	4	16	154
Medical technician	1	3	17
Lactation consultants	2	2	18
Paediatric doctors (long-term placements)	3	4	220

### Impact of nutritional intervention

Data was collected from 58 babies with birth weight < 2.5 kg before and 38 babies after training in 3 of the hospitals. There was a significant reduction in time to initiation of enteral feeds, mean 2.4 days (Standard Deviation 1.5 days) compared to 1.8 days (Standard Deviation 1.0 days), (*p* = 0.02); for time to full milk feeds (> 150 ml/kg/day of feeds or demand feeding) 10.5 days (IQR 5–14) vs 6 days (IQR 3–12) (*p* = 0.02); and for a reduction in maximum postnatal weight loss, 15% (IQR 8–20) vs 9.0% of birthweight (IQR 4.2–15), (*p* = 0.026). There was no significant difference in the number of days for birth weight to be regained (*p* = 0.22).

### Impact of respiratory support

Five CPAP machines were provided (2 financed by this grant and 3 from a separate donation) and installed in the 4 hospitals. Altogether, CPAP was used on 365 infants during the first 18 months, and was rapidly adopted by all 4 units. The most frequent complication was transient nasal or facial trauma (13%) attributed to excessive pressure applied by the cannulae in the nose or by the face mask to the nasal bridge. Significant abdominal distension was noted in 2% of infants. Pneumothorax (air adjacent to the lung), an uncommon but potentially serious complication, was not identified in any patient. As this can be difficult to identify clinically, and chest X-rays were often unavailable, this complication may have occurred but not identified. Technical problems were minor and occurred in only 2% of infants.

### Impact of revised medical records

Completeness of documentation in 30 sets of medical records after new records were introduced were compared with 30 sets before implementation. It improved in multiple areas e.g. maternal history was completed in 75% compared with 35% in previous records, all birth details in 75% compared with 44%, admission assessment of the baby in 78% compared with 53% and an admission plan in 71% compared with 51%. Daily status updates were recorded in 57%% vs 23% previously and complete prescriptions were provided in 82% of charts vs 57% previously. Growth charts, not previously available, were completed for 50% of infants at admission. Whilst all the medical staff adopted the new notes, some of the nurses continued to use their separate forms.

### Impact on thermoregulation and kangaroo mother care

The large district hospital provided KMC to 42 mothers in the year before this program, which increased to 71/year followed by 157/year during our program. In the other district hospital, following discussions with the hospital director, a room adjoining the neonatal unit was converted into a KMC room for 4 mothers.

### Impact on neonatal mortality

For the University teaching hospitals, overall mortality was reduced from 23.6% (389 deaths, 1649 admissions) in the 18 months before the project to 21.7% (463 deaths, 2135 admissions) during the 2 years of the project. For the district hospitals, overall mortality was reduced from 10% (141 deaths, 1415 admissions) to 8.1% (179 deaths, 2206 admissions). The higher mortality of the University teaching hospitals was because they were referred many high-risk mothers and preterm or sick newborn infants from other hospitals.

## Discussion

Although this was a small program, the considerable effort and intensity of training provided is shown by the many visits made by experienced doctors, neonatal nurses, lactation consultants and medical technicians from the UK. Most of the training was conducted in the neonatal unit itself and concentrated on practical aspects of neonatal care. The advantage of this training model is that through regular, short visits, intensive training can be delivered and problems dealt with without undue delay, but avoids the potential risk of trainers taking over the clinical care of the babies from local staff. The addition of some longer-term clinicians to the program allowed new initiatives to be undertaken, such as the introduction of CPAP, development of new medical records and assisting with conducting the audits, as well as reinforcing the training. However, a major issue in the implementation and adoption of improvements in care was the very small number of nurses working on each neonatal unit. This limited both nursing staff availability for training and their ability to provide additional aspects of care recommended by the partnership. Frequent staff turnover was another issue, affecting both nurses and doctors, as it necessitated repeated training of new staff and severely impeded institutional memory within the unit of new working practices. This is particularly problematic in neonatal care as new nurses have received minimal training in the care of newborn infants as part of their general nurse training. Local medical and nursing leadership and staff motivation were also major issues, aggravated by frequent moves to other departments.

This project markedly raised the profile of newborn care in the country. A Rwandan Neonatal Network was formed and meetings and workshops held for doctors and nurses from all hospitals, with contributions by visiting specialists from our program. Two of the local lead paediatricians were awarded a Royal College of Paediatrics and Child Health visiting fellowship to spend a month in a neonatal unit in the UK. However, most of the local paediatricians who provided leadership for this program are no longer working in neonatology.

For improved nutrition, we were able to introduce new feeding practices and showed significant improvement in the day of life that enteral feeds were started, the time it took to achieve full milk feeds and to reduce maximum weight loss. It was disappointing that there was no significant difference in the number of days for birth weight to be regained, but this is a more complex measure and may have needed a larger number of babies to show a significant difference; however, the reduction in maximum weight loss suggests that nutrition was improved. Posters demonstrating technique and advising on importance of expressing breast milk for preterm infants continued to be displayed on maternity and neonatal units after the end of the project, but regular reinforcement of training was required to maintain lactation support and early feeding of preterm infants.

Our experience with the introduction of CPAP highlights many of the issues in implementing new technology. Once introduced into the country, demand for CPAP rapidly increased. An additional 4 similar CPAP machines were subsequently purchased by another donor, but other new programs have resulted in different machines being introduced. Each requires its own set of guidelines and training, and different circuits and maintenance. Some, for example, do not heat or humidify the gases, making them cheaper to purchase and simpler to use; evidence to guide the choice of system according to outcomes is not available [6]. Availability of only a limited number of machines in a unit can create difficult ethical problems for staff in choosing which babies should be given CPAP, and if a machine should be transferred from one infant to another with greater need. This project was not designed to demonstrate improved outcomes from CPAP as it is standard care in high-income countries. Some authors have though argued that more evidence is needed for its use in low-income countries [7], but others disagree and consider it unnecessary [8]. Monitoring and preventing complications are clearly important, particularly tissue trauma to the nose and nasal bridge. Although this can largely be prevented by regular re-assessment and adjustment of the nasal cannula or face mask, this is much more difficult when the nurse to patient ratio is so much lower than in high-income countries. Rates of infection related to CPAP, and in particular assessment of risks of re-use of CPAP circuits and water in the humidifier could not be determined since the investigations required were not usually available. Permanent visual impairment from retinopathy of prematurity is another potential complication from delivering excessively high oxygen to preterm infants, and all infants on CPAP should have continuous monitoring of their oxygen saturation, and their inspired oxygen concentration adjusted accordingly. However, prior to the introduction of CPAP in this partnership, it was not uncommon for preterm infants with respiratory distress to be managed with high flow rates of 100% oxygen, as this was the only respiratory support available, but placed them at increased risk of retinopathy of prematurity. CPAP allows the oxygen concentration delivered to be adjusted according to need, and usually markedly reduces the oxygen concentration required, with many managed just with air. Screening for and treatment of retinopathy of prematurity is standard of care in preterm infants in high-income countries, but was unfortunately not available during this program. It is reassuring that technical problems were uncommon, being seen in only 2% of infants in this cohort. Efforts to standardise the CPAP machines and guidelines nationally and to organise purchase of replacement circuits and machine maintenance are now underway, but will require new investment and agreement between all parties involved.

The design and implementation of a new dedicated neonatal medical record produced significant improvements in documentation by doctors. However, use by nurses was inconsistent; on questioning nursing staff about this, many expressed reluctance to make entries in the same notes as the doctors. Further training and amendments to fit local agendas may be required.

Mortality rates declined during this program, which is reassuring. However, as with most quality improvement initiatives, there were many other changes occurring simultaneously to this project. In particular, towards the end of the program, the University paediatric departments had some additional paediatricians, nurses and technical support, mostly from the United States, providing long-term training as part of the HRH (Human Resources for Health) program [9]. Identifying the specific contribution made by our program was not possible, although ours was specifically neonatal and did not cover the care of older children, the main focus of other programs.

## Conclusion

This program provides an example of how health partnerships can evolve and expand from small beginnings. Some initial informal training evolved into a major program of ETAT+ courses for training in the recognition and initial management of sick children for all medical students over a period of 6 years and for health professionals in 18 hospitals. This neonatal program developed as an offshoot of that large program to address the specific needs of hospital care of newborn infants and the determination of the Ministry of Health in Rwanda to achieve its Millennium Development Goal 4 target, which was achieved [10]. It also resulted in an invitation for us and one of the University hospitals to join the WHO African Partnership for Patient Safety and the development of a further partnership to improve infection control.

This program provided a considerable amount of training from a range of neonatal health professionals. As they were all volunteers, this was done at low cost. The main immediate problems were the small numbers of staff available locally for training and high staff turnover. For long-term sustainability of the changes introduced, the main issues are local leadership, both medical and nursing, by trained staff. The number of doctors undergoing postgraduate paediatric training is slowly increasing and a small cohort of nurses are receiving some specialist neonatal training, which should help address this problem in the future. This program’s intensive training helped improve facility-based neonatal care, as documented by the audits showing better nutrition, successful introduction of respiratory support with bubble CPAP and more structured and complete medical documentation. Further improvements in neonatal and other branches of medical care could be achieved by developing similar partnership programs.

## Abbreviations

CPAP: Continuous Positive Airway Pressure
ETAT +: Emergency Triage, Assessment and Treatment plus Admission.
IQR: Interquartile Range
KMC: Kangaroo Mother Care
